# A Case of Incomplete Central Retinal Artery Occlusion Associated with Short Posterior Ciliary Artery Occlusion

**DOI:** 10.1155/2013/105653

**Published:** 2013-01-13

**Authors:** Shinji Makino, Mikiko Takezawa, Yukihiro Sato

**Affiliations:** Department of Ophthalmology, Jichi Medical University, 3311-1 Yakushiji, Tochigi, Shimotsuke 329-0498, Japan

## Abstract

To our knowledge, incomplete central retinal artery occlusion associated with short posterior ciliary artery occlusion is extremely rare. Herein, we describe a case of a 62-year-old man who was referred to our hospital with of transient blindness in his right eye. At initial examination, the patient's best-corrected visual acuity was 18/20 in the right eye. Fundus examination showed multiple soft exudates around the optic disc and mild macular retinal edema in his right eye; however, a cherry red spot on the macula was not detected. Fluorescein angiography revealed delayed dye inflow into the nasal choroidal hemisphere that is supplied by the short posterior ciliary artery. The following day, the patient's visual acuity improved to 20/20. Soft exudates around the optic disc increased during observation and gradually disappeared. His hemodynamic parameters revealed subclavian steal syndrome as examined by cervical ultrasonography and digital subtraction angiography. We speculate that his transient blindness was due to ophthalmic artery spasms. In this particular case, spasms of the ophthalmic artery and occlusion of the short posterior ciliary artery occurred simultaneously. As the short posterior ciliary artery branches from the ophthalmic artery, the anatomical location of the lesion might be near the branching of both arteries.

## 1. Introduction

There are only few reports in the Japanese literature that have presented the incomplete type of central retinal artery occlusion (CRAO), including diminished visual acuity and a residual visual field but no complete visual loss, slight retinal edema together with a slight cherry red spot on the macula, and good visual prognosis [1, 2].

The ophthalmic artery enters the orbit through the optic canal, usually inferotemporal to the optic nerve. The vessel soon crosses medially over the nerve and gives rise to its first branch, the central retinal artery. After the central retinal artery branches from the ophthalmic artery, a varying number of posterior ciliary arteries arise. Each of these major posterior ciliary arteries further divides into multiple short posterior ciliary branches that penetrate the sclera medial or lateral to the optic nerve, depending on their respective deviation from the parent medial or lateral posterior ciliary arteries [3–5].

There have been several reports presenting CRAO with choroidal circulatory disturbance [6–8], and/or anterior ischemic optic neuropathy [7, 9–11]. To our knowledge, incomplete CRAO associated with short posterior ciliary artery occlusion is extremely rare [1]. Herein, we describe the case of such a patient.

## 2. Case Report

**Figure 1:**
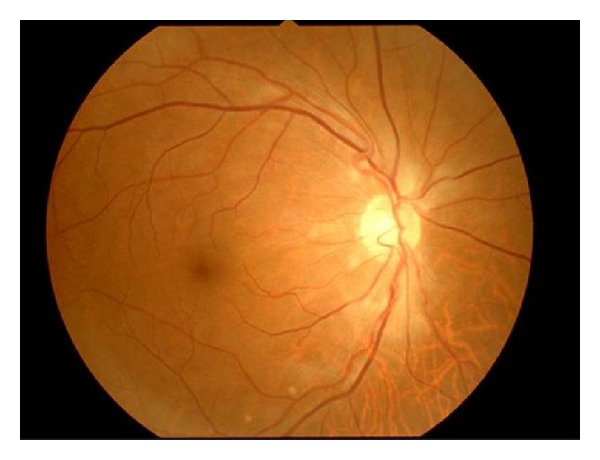
Right fundus photograph at the initial visit showing soft exudates and retinal edema around the optic disc.

**Figure 2:**
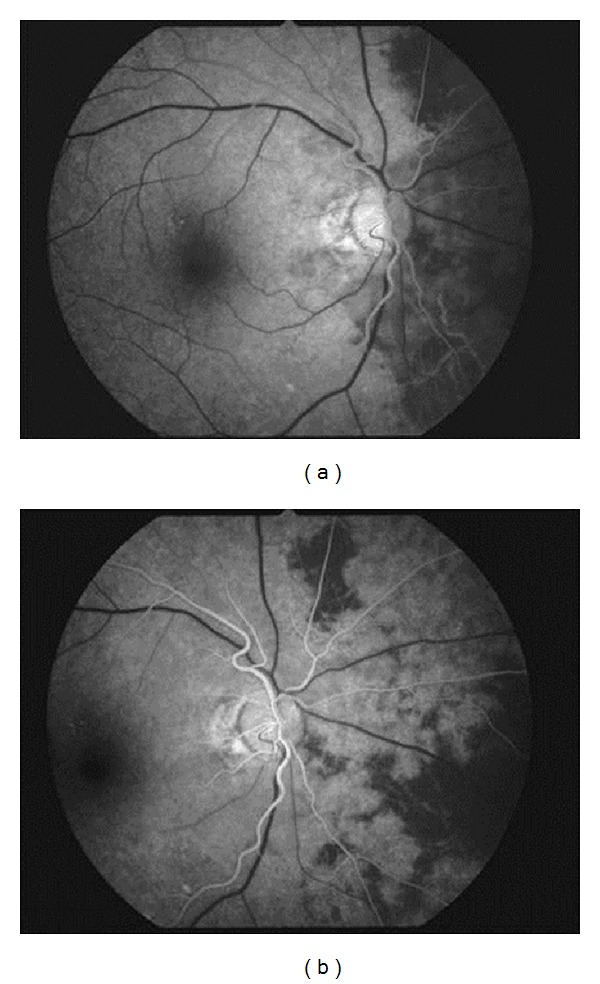
Right fluorescein angiography at the first visit demonstrated a marked filling delay of the nasal choroidal hemisphere that is supplied by the nasal short posterior ciliary artery. The hemisphere filled slowly and patchily; 27 s (a) and 30 s (b) after injection.

**Figure 3:**
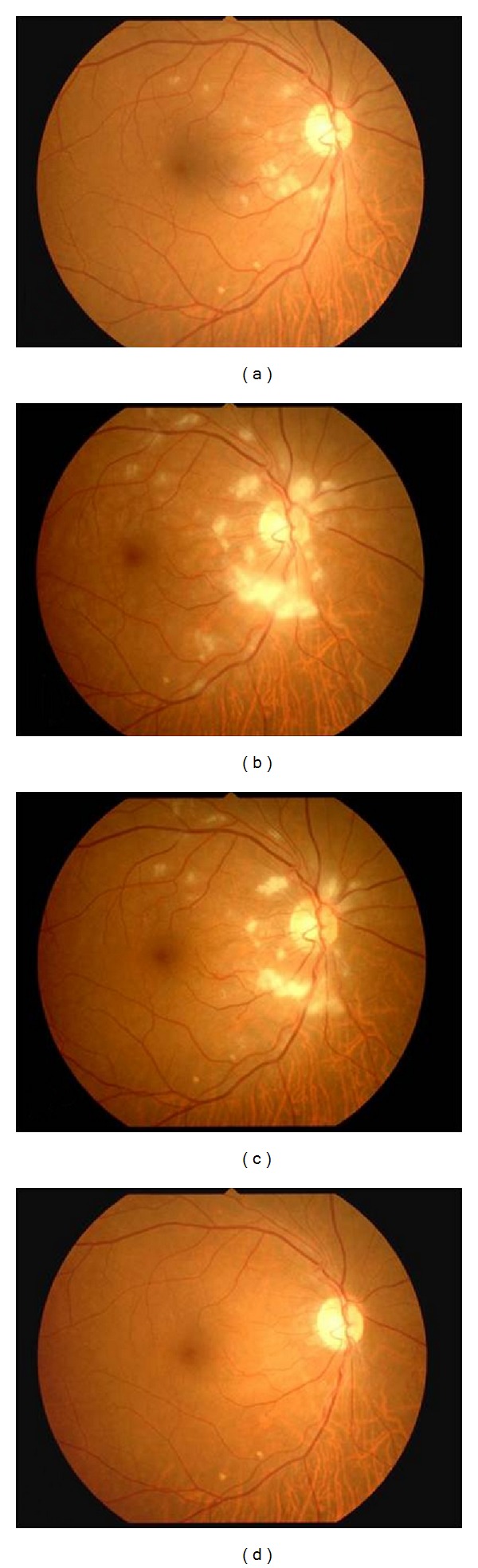
Exudate patterns on right fundus photographs after the first visit. Soft exudates increased 2 days after the initial visit (a), increased further at 1 week (b), gradually decreased at 2 weeks (c), and disappeared at 7 weeks (d).

A 62-year-old man complaining of transient blindness in his right eye on waking was referred to our hospital. The patient had a history of hypertension and hyperlipidemia. At initial examination, he had a best-corrected visual acuity of 18/20 in the right eye, which was noncorrigent, and 20/20 in the left eye. Ocular pressures were normal. Slit lamp examination showed cortical opacities in both lenses. Fundus examination showed multiple soft exudates around the optic disc and mild retinal edema in the macula of his right eye; however, a cherry red spot was not detected, and the optic disc appearance was unremarkable (Figure 1). Fluorescein angiography (FA) revealed a delay of arm-to-retina time and a marked filling delay of the nasal choroidal hemisphere that is supplied by nasal short posterior ciliary artery (Figure 2(a)). Therefore, the choriocapillaris corresponding to the nasal choroidal area filled slowly and patchily (Figure 2(b)), and no staining of the arterial wall was detected in the late stage. From these findings, the patient was diagnosed with incomplete CRAO associated with short posterior ciliary artery occlusion. Systemic administration of a vasodilator and an antiplatelet agent were started after the initial examination. On the following day, his right visual acuity improved to 20/20. However, during observation, soft exudates increased 2 days after the initial visit (Figure 3(a)), increased further at 1 week (Figure 3(b)), gradually decreased in 2 weeks (Figure 3(c)), and finally disappeared at 7 weeks (Figure 3(d)). The patient's blood pressure was 95/80 mmHg in the right arm and 130/80 mmHg in the left arm. Further examinations for evaluating hemodynamics were performed; cervical ultrasonography revealed right subclavian artery stenosis and reversed right vertebral artery flow. Additionally, digital subtraction angiography demonstrated the stenosis of the following arteries: from the right common carotid artery to the bifurcation of the internal carotid artery, the right brachiocephalic trunk, the right subclavian artery, and the left common carotid artery. On the basis of these findings, he was also diagnosed with subclavian steal syndrome. After recovery from this event, he did not experience any additional periods of transient blindness.

## 3. Discussion

In 2002, Schmidt et al. [12] classified CRAO into 3 stages; stage I of his classification represents “incomplete CRAO” and includes diminished visual acuity and a residual visual field but no complete visual loss, slight retinal edema together with a slight cherry red spot on the macula, no increase in retinal signs over several hours, and delayed but not completely interrupted blood flow revealed by FA. They also reported that spontaneous recovery usually did not occur during a followup of several hours despite minor retinal findings. The fundus changes in stage I described in their literature [12] were very similar to those in our case.

Hagimura et al. [13] evaluated 22 patients with CRAO. Eyes with poor final vision (final visual acuity < 0.1, *n* = 14) showed initially denser retinal opacities with a distinct cherry red spot. Eyes with favorable visual outcome (final visual acuity > 0.4, *n* = 7) showed soft exudates and faint retinal opacities without a cherry red spot. The findings show that the final visual outcome mainly depended on the initial visual acuity and funduscopic findings. In our patient, soft exudates were defined during observation and the patient's final visual acuity was 20/20.

There have been few reports in the Japanese literature presenting incomplete CRAO, including diminished visual acuity and a residual visual field but no complete visual loss, slight retinal edema together with a slight cherry red spot on the macula, and good visual prognosis [1, 2]. The fundus changes seen in these reported cases [1, 2] were very similar to those seen in our case. There have been several reports of CRAO presenting with choroidal circulatory disturbance [6–8], and/or anterior ischemic optic neuropathy [7, 9–11]. To our knowledge, incomplete CRAO associated with short posterior ciliary artery occlusion is extremely rare [1].

Our patient also had subclavian steal syndrome. Subclavian steal syndrome is a function of the proximal subclavian artery stenoocclusive disease with subsequent retrograde blood flow in the ipsilateral vertebral artery [14]. Morita et al. [15] described subclavian steal syndrome in a case of arteritis syndrome with bilateral occlusion of common carotid arteries. Souma et al. [16] described a case of reversed ophthalmic artery flow without occlusion of the internal carotid artery. Although, in their patient, collateral circulation and reversed ophthalmic artery flow were not blurred, stenosis of common carotid and internal carotid arteries were detected. Therefore, it is apparent that our patient had circulation disturbances in the right internal carotid artery and the right ophthalmic artery.

In conclusion, we speculate that the transient blindness experienced by our patient was due to spasms of the ophthalmic artery. In this case, spasms of the ophthalmic artery and occlusion of the short posterior ciliary artery occurred simultaneously. As the short posterior ciliary artery branches from the ophthalmic artery, the anatomical location of the lesion is likely located near the branching of both arteries.

## References

[1] Kanbara Y, Oku H, Kida T, Ikeda T (2004). A case of incomplete central retinal artery occlusion associated with ischemic optic neuropathy. *Japanese Review of Clinical Ophthalmology*.

[2] Ueda Y, Kimura T, Okamoto N, Kurimoto T, Oono S, Mimura O (2009). A case of central retinal artery occlusion with good visual acuity. *Ganka*.

[3] Onda E, Cioffi GA, Bacon DR, van Buskirk EM (1995). Microvasculature of the human optic nerve. *American Journal of Ophthalmology*.

[4] Olver JM, Spalton DJ, McCartney ACE (1994). Quantitative morphology of human retrolaminar optic nerve vasculature. *Investigative Ophthalmology & Visual Science*.

[5] Hayreh SS (1962). The ophthalmic artery. III branches. *The British Journal of Ophthalmology*.

[6] Brown GC, Magargal LE (1979). Sudden occlusion of the retinal and posterior choroidal circulations in a youth. *American Journal of Ophthalmology*.

[7] Brown GC, Magargal LE, Sergott R (1986). Acute obstruction of the retinal and choroidal circulations. *Ophthalmology*.

[8] Lieb WE, Flaharty PM, Sergott RC (1991). Color Doppler imaging provides accurate assessment of orbital blood flow in occlusive carotid artery disease. *Ophthalmology*.

[9] Hayashi K, Wasano T, Ohnishi Y (1984). A case of anterior optic neuropathy due to occlusion of the left nasal short posterior ciliary artery. *Folia Ophthalmologica Japonica*.

[10] Itoi K, Sugasawa J, Ikeda T (2005). Retarded dye inflow in the nasal choroidal hemisphere in a case of central retinal artery occlusion with anterior ischemic optic neuropathy. *Japanese Journal of Clinical Ophthalmology*.

[11] Okamoto N, Kurimoto T, Mimura O (2007). A case of simultaneous central retinal artery occlusion and choroidal circulation disturbance. *Journal of the Eye*.

[12] Schmidt DP, Schulte-Mönting J, Schumacher M (2002). Prognosis of central retinal artery occlusion: local intraarterial fibrinolysis versus conservative treatment. *American Journal of Neuroradiology*.

[13] Hagimura N, Kishi S, Iida T (1994). Clinical manifestations and visual outcome in central retinal arterial occlusion. *Japanese Journal of Clinical Ophthalmology*.

[14] Osiro S, Zurada A, Gielecki J, Shoja MM, Tubbs RS, Loukas M (2012). A review of subclavian steal syndrome with clinical correlation. *Medical Science Monitor*.

[15] Morita S, Souma N, Amano Y (2002). Absence of ocular ischemia in a case of arteritis syndrome with bilateral occlusion of common carotids. *Japanese Review of Clinical Ophthalmology*.

[16] Souma N, Tasaka Y, Nakauchi K, Kubota Y, Amano Y, Sogabe T (2000). A case of reversed ophthalmic artery flow without occlusion of the internal carotid artery. *Journal of Japanese Ophthalmological Society*.

